# Anisotropy of Transport Properties Correlated to Grain Boundary Density and Quantified Texture in Thick Oriented Ca_3_Co_4_O_9_ Ceramics

**DOI:** 10.3390/ma11071224

**Published:** 2018-07-17

**Authors:** Driss Kenfaui, Moussa Gomina, Jacques Guillaume Noudem, Daniel Chateigner

**Affiliations:** 1 Université Toulouse 3-Paul Sabatier, LAPLACE-UMR CNRS 5213, CIRIMAT-UMR CNRS 5085, 118 Route de Narbonne, CEDEX 9, 31062 Toulouse, France; 2 Normandie Université, CRISMAT-UMR 6508 CNRS/ENSICAEN, 6 Bd Maréchal Juin, CEDEX 04, 14050 Caen, France; moussa.gomina@ensicaen.fr (M.G.); jacques.noudem@ensicaen.fr (J.G.N.); daniel.chateigner@ensicaen.fr (D.C.)

**Keywords:** Ca_3_Co_4_O_9_ thermoelectric oxides, spark plasma sintering/texturing, combined analysis, crystallographic texture, grain boundary density, transport properties, anisotropy

## Abstract

The misfit-layered Ca_3_Co_4_O_9_ oxide is being seen as a potential thermoelectric (TE) candidate for high-temperature power generation in air. Given the very small size and low strength exhibited by single crystals, grain-oriented Ca_3_Co_4_O_9_ ceramics are worth elaborating to capitalize on their anisotropy. However, the usual textured pellets are too thin to probe the TE properties along their principal crystallographic directions. In this paper, we report on the anisotropy of TE properties in the 350–860 K range within thick textured Ca_3_Co_4_O_9_ ceramics fabricated by moderately pressing at 1173 K stacks of pellets primarily textured using spark plasma sintering (SPS), spark plasma texturing (SPT), and hot pressing (HP). The texture was quantitatively assessed, and the influent microstructural parameters were identified, particularly the grain boundary density parallel (GBD^c^) and perpendicular (GBD^ab^) to the mean c*-axis. We found that the edge-free processing fostered material texturing and (a,b) plane grain growth, thereby dropping GBD^ab^ and increasing GBD^c^. This resulted in a resistivity *ρ*^ab^ reduction, leading to a marked enhancement in power factor *PF*^ab^, which reached 520 μW·m^−1^·K^−2^ at 800 K for the HP sample. The anisotropy *ρ*^c^/*ρ*^ab^ was substantially promoted as the texture was reinforced and the GBD^c^/GBD^ab^ ratio increased, with *ρ*^c^/*ρ*^ab^ (_HP_) > *ρ*^c^/*ρ*^ab^ (_SPT_) > *ρ*^c^/*ρ*^ab^ (_SPS_). The Seebeck coefficient *S* also revealed an anisotropic behavior, with a ratio S^c^/S^ab^ >1 for the SPS-processed materials. This behavior was reversed (S^c^/S^ab^ <1) for the more textured SPT and HP specimens. It therefore resulted in a PF anisotropy *PF*^c^/*PF*^ab^ (_HP_) < *PF*^c^/*PF*^ab^ (_SPT_) < *PF*^c^/*PF*^ab^ (_SPS_). The *PF*^ab^/*PF*^c^ ratio attained 13.6 at 800 K for the thick HP sample, which is the largest ratio recorded thus far on undoped Ca_3_Co_4_O_9_ ceramics.

## 1. Introduction

Thermoelectric (TE) materials, which have the ability to directly convert between thermal and electrical energy, offer a unique solution to sustainable power generation from various waste heat sources [1,2,3]. Their performance is defined by the dimensionless figure of merit (*ZT)* and expressed as *ZT* = *S^2^T*/*ρκ,* where *S*, *ρ*, *κ* and *T* are the Seebeck coefficient, electrical resistivity, thermal conductivity, and absolute temperature, respectively [3]. While most high-performance TE compounds depict chemical and thermal instabilities in air and comprise scarce, expensive, or toxic elements [4,5,6,7,8,9,10,11,12,13], metal oxides are seen as an alternative to surmount these shortfalls and have therefore received increasing attention in the last two decades [14,15,16,17,18,19,20,21,22,23]. A great deal of effort has gone into not only discovering new compounds, but also assessing the TE performance of existing bulk oxides materials, leading to an encouraging progress. Polycrystalline ZnO and In_2_O_3_ *n*-type oxides have been found with marked *ZT* values as high as 0.4 and 0.45 at 1247 K, respectively [19,20]. The polycrystalline Ca_3_Co_4_O_9_ system has been reported to be one of the best *p-*type oxides for TE energy conversion applications at high temperature because it does not present the aforesaid shortfalls and exhibits good TE properties, leading to *ZT* values of 0.2–0.4 in the 950–1100 K range [15,17,21,22,23]. Ca_3_Co_4_O_9_ crystallizes in a misfit-layered structure, which consists of CdI_2_-type triangular CoO_2_ lattice and a layered rock salt part of three Ca_2_CoO_3_ units alternatively stacked along the c-axis [15]. Some or all of its TE properties are hence anisotropic, thereby fostering TE performance following a specific crystallographic direction. To capitalize on such anisotropic character, grain-oriented Ca_3_Co_4_O_9_ ceramics are worth elaborating [24], especially as single crystals would be too expensive to grow and shape. They also have a very small size and poor mechanical strength, which makes them unsuited for consideration in practical applications. For that purpose, we have explored in recent works [25,26,27,28] the spark plasma sintering (SPS) method with the aim of processing textured Ca_3_Co_4_O_9_ ceramic pellets toward the promotion of current flow in their in-plane (i.e., (a,b) planes) and, subsequently, reducing the corresponding electrical resistivity *ρ*^ab^. This was shown to result in an enhancement of the power factor *PF*^ab^ = S^ab2^/*ρ*^ab^ compared to the naturally sintered (NS) ceramics. However, the achieved *PF*^ab^ values remained significantly lower than the ones we reported for Ca_3_Co_4_O_9_ ceramics textured by the hot pressing (HP) process, which was mainly derived from a stronger texture and a larger (a,b) plane mean grain size that yielded much more *ρ*^ab^ reduction [24,28]. Indeed, the grain growth and orientation were experimentally shown to be limited when using the SPS process, imputable to the lateral pressure applied by the mould walls on the material causing an antagonist effect to the uniaxial pressure. To overcome this barrier, we used an amended SPS processing—referred to as edge-free spark plasma sintering or spark plasma texturing (SPT)—enabling free deformation and orientation of the grains, and thus inducing a rapid material texturation [29]. Even so, the SPT-processed Ca_3_Co_4_O_9_ pellets, as is the case with HP-processed ones, were too thin (thickness below 0.7 and 0.5 mm, respectively) [24,27,28,29] to probe the TE properties along their principal directions for anisotropy investigations and to design TE ceramic elements with required sizes for the development of TE devices.

In this paper, we report a thorough study of the anisotropy of transport properties probed parallel and perpendicular to the mean c*-axes of thick textured Ca_3_Co_4_O_9_ ceramics obtained by moderately pressing at 1173 K stacks of samples originally textured using SPS, SPT, and HP methods. The crystallographic texture was quantitatively investigated using combined analysis, and the influent microstructural parameters were determined. The temperature dependence of the electrical resistivity *ρ* and Seebeck coefficient *S* were probed in the 350–860 K range, parallel (*ρ*^c^; *S*^c^) and perpendicular (*ρ*^ab^; *S*^ab^) to the applied pressing axis. The induced anisotropy of transport properties as well as of power factor was correlated to the texture strength and the grain boundary density and compared to that of the reported textured materials.

## 2. Fabrication of Textured Ca_3_Co_4_O_9_ Ceramic Stacks

**Figure 1:**
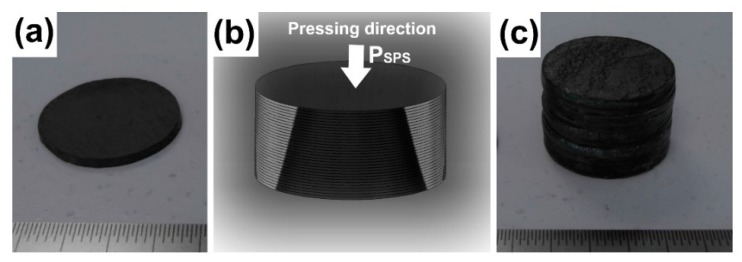
Fabrication of thick Ca_3_Co_4_O_9_ ceramics with spark plasma sintering (SPS) and spark plasma texturing (SPT) processes. (**a**) SPS-processed single pellet with 2 mm in thickness. (**b**) Schematic of a batch of single mirror-polished SPS pellets stacked along their mean c*-axis. (**c**) A ~10 mm-thick sample obtained upon the treatment of the SPS stack at 1173 K under a moderate pressure of 28 MPa for 10 min. The SPT thick sample was elaborated similarly to the SPS one, but the single textured pellets with 0.7 mm in thickness were initially prepared in an edge-free mould.

The Ca_3_Co_4_O_9_ powders were synthesized by high temperature solid-state reactions from CaCO_3_ and Co_3_O_4_ commercial precursors weighted in the appropriate stoichiometric ratios [24]. The synthesis involved mixing these starting powders in an agate ball mill until getting a homogenous mixture and calcining it in air at 1173 K for 24 h to purify the Ca_3_Co_4_O_9_ phase by decomposing the carbonates. The textured Ca_3_Co_4_O_9_ ceramic stacks were then fabricated as described below:

(i) A 2 mm-thick pellet (Figure 1a) was prepared using the SPS process as follows: The Ca_3_Co_4_O_9_ powder was poured into a graphite mould with an inner diameter of 13 mm. A pulsed electric current (2500 A, 4 V) was injected through the mould to heat the material up to the dwell temperature T_SPS_ = 1173 K while keeping it under a uniaxial pressure P_SPS_ = 50 MPa for t_SPS_ = 2 min under vacuum (10^−3^ bar).

A batch of single SPS pellets were mirror-polished and stacked along their mean c*-axis, as illustrated in Figure 1b, without using any sintering agent. This arrangement was then treated at 1173 K for 10 min under a moderate pressure of 28 MPa to obtain a stack thickness of about 10 mm, as presented in Figure 1c.

(ii) Using the SPT process, a 0.7 mm-thick sample was elaborated similarly to the SPS one but with an edge-free mould. First, the Ca_3_Co_4_O_9_ powder was cold-compacted in a 13 mm-diameter mould and naturally sintered at 1173 K for 2 h. The obtained preform was then placed in the centre of a graphite mould with a larger diameter of 20 mm and was made to undergo the same experimental conditions (T_SPS_, P_SPS_, t_SPS_) applied in the case of the single SPS samples. The idea here was to allow free deformation and orientation of the grains with the purpose of inducing a prominent grain growth and texture, respectively.
  A batch of single SPT-processed pellets were mirror-polished and also stacked along their mean c*-axis before being treated in the same conditions as the SPS stack.
(iii) On the other hand, 0.5 mm-thick samples were prepared using the HP process. The Ca_3_Co_4_O_9_ powder was first cold-compacted into 4 mm-thick and 25-mm diameter pellets. The pellet was set in a homemade furnace between two silver foils to avoid an undesirable reaction with the alumina bearing plates. The sample was then heated to the dwell temperature of P_HP_ = 1193 K and maintained for t_HP_ = 24 h under a uniaxial pressure P_HP_ = 30 MPa under air atmosphere.

**Figure 2:**
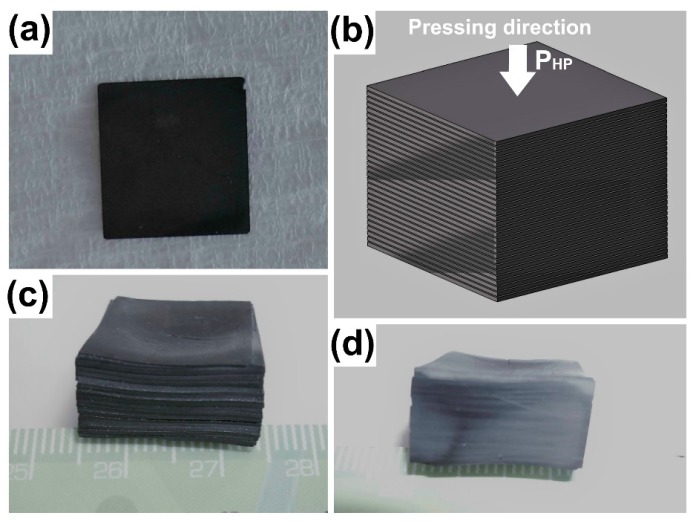
Fabrication of thick Ca_3_Co_4_O_9_ ceramics using the hot pressing (HP) process. (**a**) HP-textured single sample cut into ~18 × 18 × 0.5 mm^3^ parallelepiped form and mirror-polished. (**b**) Schematic of a batch of single HP samples stacked along their mean c*-axis. (**c**) A ~9 mm-thick sample obtained upon the treatment of the HP stack at 1193 K under a moderate uniaxial pressure of 10 MPa for 10 h. (**d**) Cross-section view upon cutting the HP stack in the direction parallel to the pressing axis.

The obtained HP pellets were cut into ~18 × 18 × 0.5 mm^3^ parallelepiped single samples, which were then mirror-polished on their two faces (Figure 2a). They were stacked together along their mean c*-axis, as can be seen in Figure 2b, and HP-treated at 1193 K under a moderate uniaxial pressure of 10 MPa for 10 h to achieve a sample with a thickness as high as 9 mm (Figure 2c,d). Note that the total time for the SPT stack processing was about 15 h, whereas the time taken for the HP stack exceeded 1100 h.

## 3. Quantified Crystallographic Texture and Microstructure

X-ray diffraction was performed using a four-circle diffractometer in order to attest the presence of the sole Ca_3_Co_4_O_9_ phase in the specimens and to investigate and quantify sample textures. This diffractometer was equipped with a curved position sensitive detector (CPS120 from ThermoScientific, Waltham, MA, USA), which operates with a monochromatized Kα-Cu radiation [30] within the combined analysis formalism [31] implemented in the Material Analysis Using Diffraction (MAUD) software [32]. The approach taken here [24] was based on quantifying the texture from cyclic Rietveld refinement of 13 diagrams measured every 5° in tilt angle *χ* (sample orientation) at an incident angle of the X-ray beam on the sample of *ω* = 20°.

The recalculated pole figures were normalized into orientation densities, expressed in multiples of a random distribution (mrd) unit. Therefore, a sample without preferred orientations or starting powder displayed uniform pole figures with 1 mrd levels, but a textured material showed pole figures with minima and maxima of orientation densities spanning from 0 mrd (revealing the absence of crystals oriented in this direction) to infinity as in the case for a single crystal along few directions. These normalized pole figures were computed from the orientation distributions (OD) of crystallites, refined using the E-WIMV formalism [33] after extraction of the peak intensities during the Rietveld cycles. The Ca_3_Co_4_O_9_ supercell definition [34] was used for these refinements and a sample reference frame was chosen so that the direction of pressures (P_SPS_ and P_HP_) application corresponds to the centre of the pole figures.

The 2θ diagrams recorded for every χ-orientation of SPS, SPT, or HP sample showed a decrease of the 00L lines when inclining it from χ = 0° (bottom) to χ = 60° (top), which reflected a preferred orientation component with the c*-axes preferentially aligned with the applied pressure axis (χ = 0°) in these materials. A quantitative comparison between samples in terms of the grain-orientation degree could not be directly made from these 2θ diagrams as they simply showed diffracted intensities. However, by using combined analysis, the diagram fits enabled the extraction of the parameters required for such comparison. The comparison between the calculated diagrams and the experiments yielded a reproducibility considered between satisfactory, for the SPS textured-sample, and good in the case of the HP one for which the reliability factors are as low as *Rw* = 3.26%, *R_Bragg_* = 2.27%, *R_expected_* = 0.85%, giving a goodness of fit *GoF =* 3.81. The resulting cell parameters after refinement were *a* = 4.8573(3) Å, *b* = 36.526(3) Å, *c* = 10.8547(3) Å, *β =* 97.913(3)°, in accordance with the typical bulk values for the Ca_3_Co_4_O_9_ phase [15].

**Figure 3:**
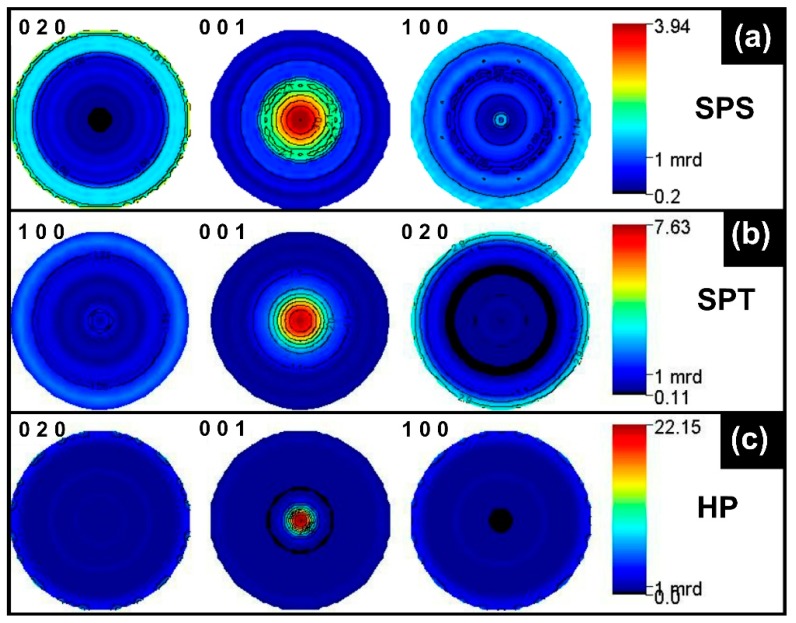
{100}, {001}, and {010} pole figures achieved after the combined refinement of the orientation distribution function (ODF) for the (**a**) SPS, (**b**) SPT, and (**c**) HP samples.

**Figure 4:**
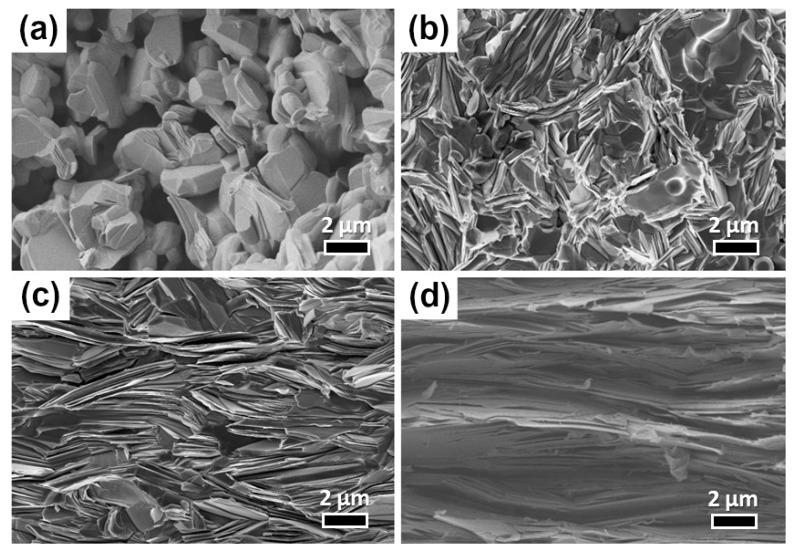
SEM micrographs of fractured surfaces parallel to the applied pressing axis of the Ca_3_Co_4_O_9_ samples processed by (**a**) naturally sintered (NS), (**b**) SPS, (**c**) SPT, and (**d**) HP processes.

The refinement of the orientation distribution (OD) allowed the reconstruction of the {020}, {001}, and {100} pole figures of the SPS, SPT, and HP samples given in Figure 3. The crystallographic texture of the SPT sample, signed by a maximum of 7.6 mrd on the {001} pole figure (Figure 3b), was two times stronger compared to the classical SPS one (Figure 3a); this was in line with the scanning electron microscope (SEM) observations undertaken on surfaces of rupture in the (a,b) planes using a Carl Zeiss (Supra 55, Oberkochen, Germany) SEM. Indeed, while the SPS sample exhibited close-packed platelets arranged in aggregates with no clear preferred orientation except in small zones where a grain-alignment could be distinguished (Figure 4b), the SPT sample depicted a homogeneous microstructure with distinctly oriented aggregates with their planes perpendicular to the pressing direction (Figure 4c). Therefore, the SPT processing occurred with a manifest transformation in terms of grains morphology, freely deformed into platelets displaying a larger (a,b) plane length (≤14 μm) compared to SPS (≤8 μm). This material deformation was achievable due to the available space in the larger mould enabling platelets’ lateral growth concomitant with crystallite rotation towards an alignment of their c*-axes parallel to the pressing axis. Both samples displayed similar densities of about 98%, which was assessed by comparing the value measured via the Archimedes method (KERN & Sohn GmbH, Baligen, Germany) to the theoretical one [15]. Note that the achieved density was drastically larger compared to that recorded for the naturally sintered (NS) sample (Figure 4a).

Apart from the <001>* fiber texture (Figure 3), the SPT sample did not show any significant minor orientation component, as can be seen from the {100} and {010} pole figures (Figure 3b). This was unlike the SPS sample in which small <010> fiber orientation component existed with <010> parallel to the SPS pressing axis (Figure 3a). Consequently, the SPT sample exhibited a larger texture strength compared to SPS, with nearly twice the maximum orientation density for the centre of the {001} pole figure. However, its texture remained weaker than that of the HP sample, which displayed a maximum of the {001} poles around 22 mrd. This is the largest value achieved hitherto on Ca_3_Co_4_O_9_ bulk materials. The pole figures noticeably showed that the <001>* directions were strongly aligned parallel to the pressing axis. This was further supported by the SEM micrograph of fractured surfaces containing the c*-axis of the HP sample (Figure 4d), which showed a 96% densified and homogeneous microstructure that showed a much larger orientation degree with more pronounced platelets growth (≤17 μm), compactly stacked up along the HP pressing axis. Note that the SPS, SPT, and HP techniques induced a severe material densification compared to NS (Figure 4a).

From the results, it can be seen that even though the HP technique enabled much stronger textures compared to SPT, the latter offers a new way to process lamellar ceramics like Ca_3_Co_4_O_9_ with quicker material texturing, thereby bringing outstanding time and energy savings. However, further investigations using SPT—especially in terms of optimizing the pressure and temperature cycles as well as the diameter of the used mould—are needed for further texture reinforcement. For instance, less than 2 h of total elaboration time was needed in SPT to achieve such observed orientation and platelet lateral dimension (compared to 36 h in HP). The effect of larger SPT times have not been tested in the present work, but it is possible that a further increase in both platelets alignment and size can be achieved by a moderate increase in dwell time.

## 4. Anisotropy of Transport Properties

**Figure 5:**
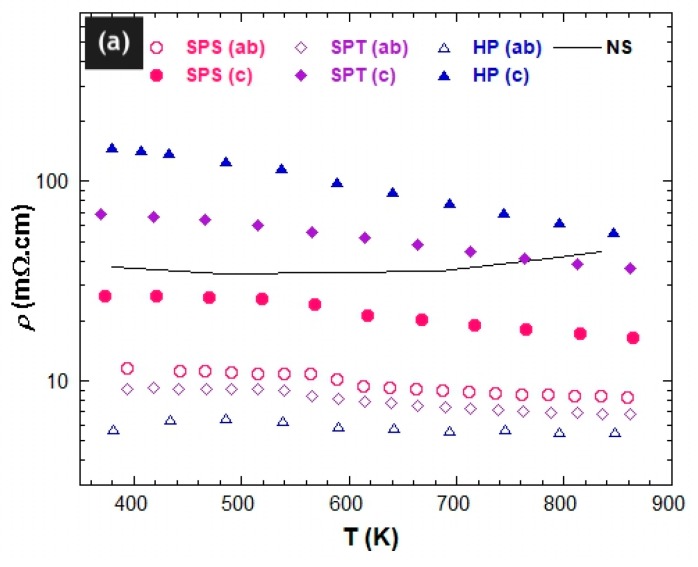

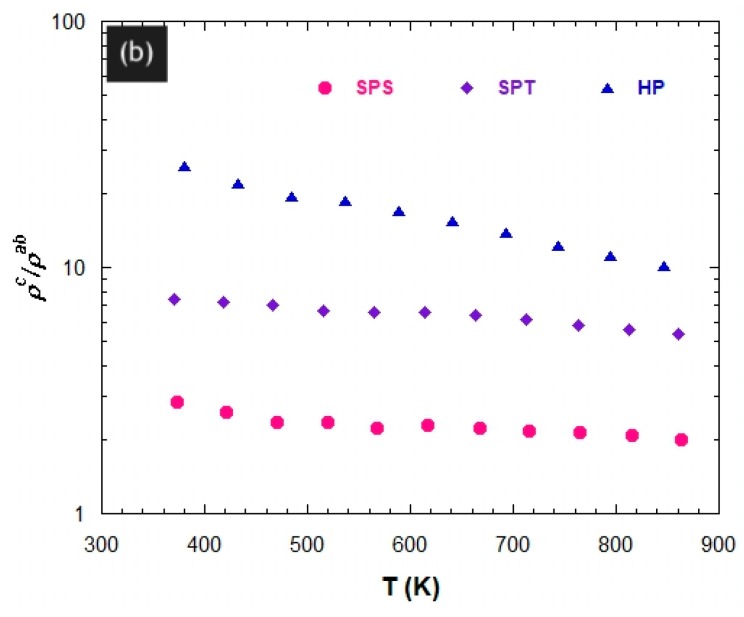
(**a**) Temperature dependence of the electrical resistivity measured on ~2 × 2 × 8–10 mm^3^ bars cut from the fabricated SPS, SPT, and HP stacks along their two principal directions: parallel (*ρ*^c^) and perpendicular (*ρ*^ab^) to the applied pressing axis. The *ρ*^NS^ (T) recorded for the NS sample is also given for easy comparison. (**b**) Temperature dependence of the anisotropy in electrical resistivity of the SPS, SPT, and HP samples.

The temperature dependence of the electrical resistivity *ρ* and of the Seebeck coefficient *S* was monitored in the 350–900 K range using a ZEM-3 apparatus (ULVAC-RIKO, Inc., kanagawa, Japan). These properties were measured on ~2 × 2 × 8–10 mm^3^ bars cut from the fabricated stacks along their two principal directions: parallel (*ρ*^c^; *S*^c^) and perpendicular (*ρ*^ab^; *S*^ab^) to the applied pressing axis. Figure 5a shows the electrical resistivity curves *ρ*(T) measured along these two main directions for thick textured SPS, SPT, and HP samples. The *ρ*^NS^(T) curve recorded for the naturally sintered Ca_3_Co_4_O_9_ ceramic is also given for comparison. The *ρ*(T) curves exhibited a transition around 560 K reported to be assigned to either magnetic or structural transition [15,35], and this behavior was present for both measured directions irrespective of the texturing method used. Therefore, we noted two regions where the *ρ*^c^(T) curves indicated first a slight decrease in transport behavior from 350 to 560 K, then a more pronounced decrease from 560 K up to 860 K, reflecting a semiconducting behavior. Noticeably, the *ρ*^c^(T) transition around 560 K was more visible for the lowest textured samples; the SPS sample exhibited a more marked step compared to just a slope variation for both SPT and HP. The *ρ*^ab^ (T) curves also exhibited a similar step but more pronounced. In addition, a *ρ*^ab^ (T) metallic behavior was observed. This varied from up to nearly 500 K in HP, to below 400 K in SPT; for SPS, the value was not visible in our temperature range. Consequently, the resistivity behavior of our samples strongly depended on the achieved texture strength for both the anisotropy and metallicity. As usual, for strongly anisotropic crystal structures like Ca_3_Co_4_O_9_, stronger textures gave rise to larger resistivity anisotropies of the samples. However, because this phase also exhibited a different resistive behavior along c- and a-axes [15], their respective signatures were also visible in our textured samples. In the strong-texture SPT and HP, the metallic behavior was observed in *ρ*^ab^(T) and not in *ρ*^c^(T), as only the (a,b) planes exhibited such metallicity in single crystals. In the low-texture SPS sample, which behaves like a random powder, the averaging over crystal orientations masked the metallic character down to 400 K. However, the peculiar behavior around 560 K has never been reported in the literature, and our measurements also show that the latter is texture-dependent, i.e., associated to the (a,b) planes. The SPS, SPT, and HP samples presented much lower *ρ*^ab^ values compared to *ρ*^c^ (as also observed on single crystals) [15] and to the NS sample. The comparison with NS was largely ascribed to larger densification (>96%) of the textured materials, which also explains why *ρ*^c^_SPS_(T) < *ρ*_NS_(T) in the entire temperature range. Comparing SPS, SPT, and HP materials, the respective *ρ*^ab^ decrease was principally tied to the texture strength and the (a,b) plane grain length (*ℓ*^ab^), which determined the grain boundaries density (GBD^ab^) along the direction perpendicular to the pressing axis (mean (a,b) planes). The stronger the texture and/or lower the GBD^ab^, the lower the *ρ*^ab^ values. Their respective *ρ*^ab^ values at 800 K were 8.5, 6.7, and 5.3 mΩ·cm corresponding to ~5, 6, and 8 times lower than the NS sample (42 mΩ·cm), respectively. The resistivity *ρ*^ab^ of the HP-textured sample approached the values recorded in the single crystal (~3.8 mΩ·cm at 800 K) [15,36]. It was also close to the values reported for thin films by Sugiura et al. (~3.4 and ~3.9 mΩ·cm at 300 and 800 K, respectively) [37,38] and was even lower than the values published by Sakai et al. (~8 mΩ·cm at 300 K) [39]. This result reflects the role of the achieved texture and the low GBD^ab^ in achieving the electrical conduction in the (a,b) planes of the oriented Ca_3_Co_4_O_9_ ceramics.

The gap between *ρ*^ab^(T) and *ρ*^c^(T) curves showed a manifest anisotropy (Figure 5b) arising primarily from the texture, which promoted electrical current flow in the (a,b) planes inducing a *ρ*^ab^ reduction. Looking at the values of the maximum orientation densities in the {001} pole figures (Figure 3), this effect was more pronounced in the SPT sample than in SPS but even more marked in the HP sample. Consequently, even when the current was injected parallel to the pressure axis (mean c*-axes), it partially circulated in the (a,b) planes. This partly contributed to the electrical conduction in these planes, thereby causing the resistivity difference between *ρ*^ab^ and *ρ*^c^ to increase with the texture strength. However, we cannot exclude the *ρ*^ac^ (i.e., *ρ*^13^) tensor component contribution as it is not a priori zero (although it has never been measured on single crystals) in the Ca_3_Co_4_O_9_ monoclinic system [40]. The resistivity anisotropy could have also come from the lower GBD^ab^ contribution compared to the grain boundary density in the planes containing the c*-axis (GBD^c^). Indeed, Ca_3_Co_4_O_9_ crystallizes as platelets (of length *ℓ*^ab^) with the smaller dimension (thickness) *e*^c^ along c*, particularly in textured materials. This resulted in a larger GBD^c^ along the pressure axis in our materials compared to perpendicularly, inducing a smaller *ρ*^ab^ and a larger *ρ*^c^. Because the grain dimensions *ℓ*^ab^ and *e*^c^ measured within the SPS, SPT ,and HP samples were found as *ℓ*^ab^ (_SPS_) < *ℓ*^ab^ (_SPT_) < *ℓ*^ab^ (_HP_) and *e*^c^ (_SPS_) > *e*^c^ (_SPT_) > *e*^c^ (_HP_), their grain boundary densities obeyed GBD^ab^ (_SPS_) > GBD^ab^ (_SPT_) > GBD^ab^ (_HP_) and GBD^c^ (_SPS_) < GBD^c^ (_SPT_) < GBD^c^ (_HP_), leading to GBD^c^/GBD^ab^ (_SPS_) < GBD^c^/GBD^ab^ (_SPT_) < GBD^c^/GBD^ab^ (_HP_). The obtained GBD^c^/GBD^ab^ associated to the texture strengths achieved for SPS, SPT, and HP, respectively, explains the electrical resistivities (depicted in Figure 5a) that led to the anisotropy ratio *ρ*^c^/*ρ*^ab^ (_HP_) > *ρ*^c^/*ρ*^ab^ (_SPT_) > *ρ*^c^/*ρ*^ab^ (_SPS_). The *ρ*^c^/*ρ*^ab^ ratio reached 2, 5.8, and 10.6 at 800 K for these samples, respectively. Resistivity measurements by Masset et al. [15] on the single crystal and by Sakai et al. [39] on thin films indicate an anisotropy *ρ*^c^/*ρ*^ab^ ratio of 17 and 40 at 300 K, respectively, which can explain the observed HP anisotropy with 22 mrd of orientation density (Figure 3). However, for the other two samples, texture alone cannot account for the resistivity anisotropy signature, and grain boundaries also play a significant role. In the absence of single crystal anisotropic measurements for larger temperatures, any quantitative simulation of the macroscopic properties of our samples was not possible.

**Figure 6:**
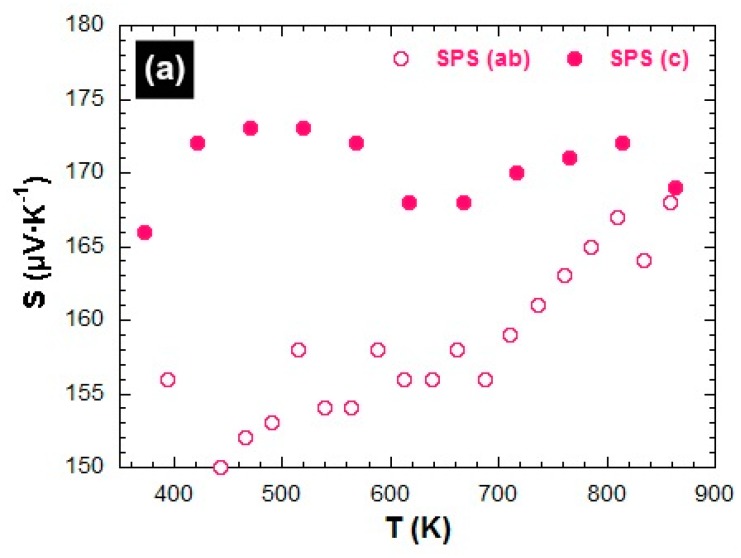

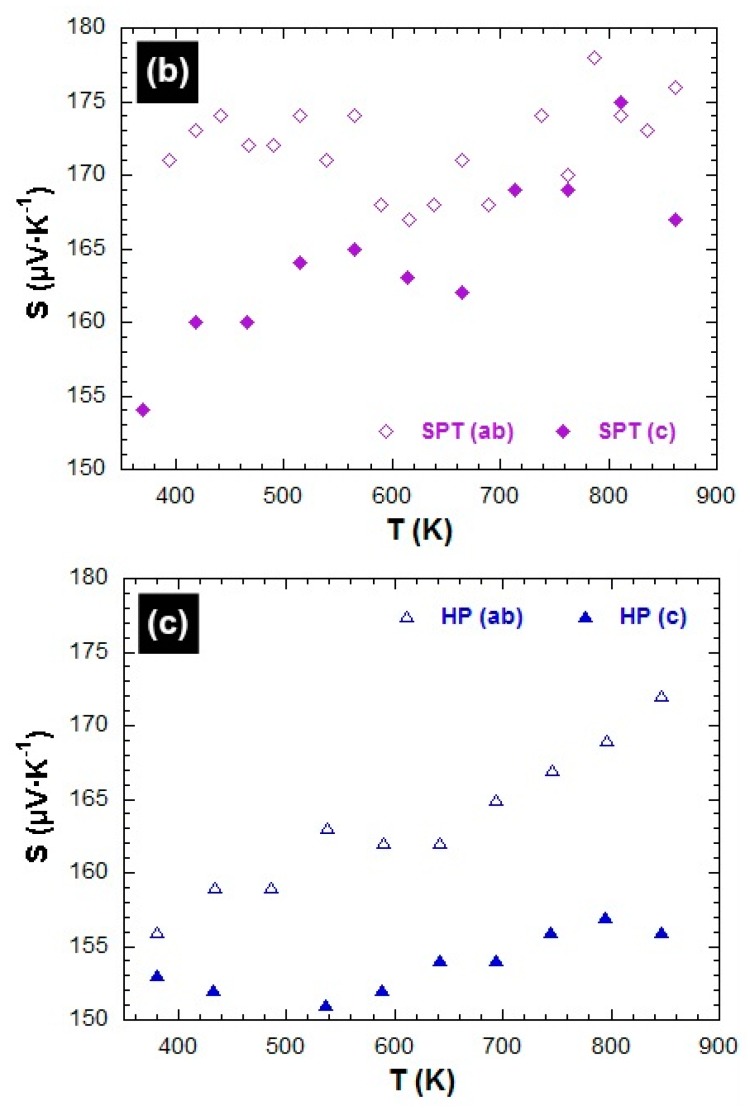
Temperature dependence of the Seebeck coefficient measured parallel (S^c^) and perpendicular (S^ab^) to the mean c*-axis (**a**) SPS, (**b**) SPT, and (**c**) HP stacks.

Figure 6a–c shows the temperature dependence of the Seebeck coefficient measured parallel (S^c^) and perpendicular (S^ab^) to the mean c*-axis of the SPS, SPT, and HP stacks, respectively. The S(T) curves also show anisotropy between S^ab^ and S^c^, which is in agreement with the measurements reported by Tang et al. [41] and Schrade et al. [42]. It fits the thermopower description with second-order tensors [40,43] written as S^m^_ij_. This description has not yet been experimentally determined for Ca_3_Co_4_O_9_ single crystals or for ceramics to the best of our knowledge. For the monoclinic Ca_3_Co_4_O_9_ point group, the single crystal Seebeck coefficient tensor is defined with five independent components as S^m^_12_ = S^m^_21_ = S^m^_23_ = S^m^_32_ = 0. In the textured bulk ceramics that have a <001>* fiber texture, there are only two independent components as S^m^_11_ = S^m^_22_ and S^m^_31_ = S^m^_13_ = 0 arising from the macroscopic symmetry in the ∞m Curie group. Therefore, we measured S^m^_11 =_ S^ab^ and S^m^_33_ = S^c^ for the textured samples.

Like the electrical resistivity, the Seebeck coefficient curves exhibited a transition around 540–600 K depending on the sample, and a thermopower anisotropy that appeared to be influenced by the texture strength and/or the grain boundary density ratio, GBD^c^/GBD^ab^. In the low textured material (SPS sample), the difference between S^c^(T) and S^ab^(T) values was larger in the 350–560 K than in the 560–860 K range (Figure 6a). Although the S^c^/S^ab^ ratio was larger than 1 for all the measured temperature range, it decreased with temperature and reached 1 at 860 K for this sample. The thermopower anisotropy behavior was reversed (S^c^/S^ab^ < 1) as the texture strength was reinforced and the GBD^c^/GBD^ab^ ratio increased. Indeed, in the ceramic displaying an intermediate texture strength level (SPT sample), the anisotropy ratio was found S^c^/S^ab^ < 1 in the 350–560 K range and close to 1 for the transition temperature (Figure 6b). This ratio was clearly S^c^/S^ab^ < 1 along all the measuring range for the most textured material (HP sample, Figure 6c), in accordance with the thermopower values reported by Tang et al. [41] and Schrade et al. [40] on Ca_3_Co_4_O_9_ ceramics. For the latter sample, the difference between the two measured directions even increased with temperature. From Figure 3c, it would be tempting to infer a much lower S_33_ single crystal Seebeck tensor component compared to S_11_ and S_22_, giving rise to a much lower S^c^ at a macroscopic scale because of the strong sample texture. However, if this was the case, it would not explain the S^ab^ and S^c^ reverse behavior that we observed in the lower texture strength samples (Figure 6a,b), which should not have exhibited S-anisotropy. We therefore conclude that in our samples, the S-anisotropic behavior mainly originated from grain and crystallite boundaries anisotropy, even though we cannot completely exclude small intrinsic anisotropy of the S_ii_ tensor components in the constituting single crystals from our measurements. However, if any anisotropy of S_ij_ of the Ca_3_Co_4_O_9_ crystal did occur, this could not have been larger than a few percent. This interpretation was reinforced by the temperature behavior of S^ab^ and S^c^. Here, S^ab^ showed a large variation depending on the temperature, while S^c^ was rather constant at 5–7% on the whole temperature range. In addition, S^ab^ increased with T, indicating that GDB^ab^ was the dominant cause of Seebeck anisotropy.

By contrast, the S^ab^ seemed to decrease as the texture was reinforced and the GBD^ab^ diminished, particularly at high temperatures. S^ab^ went from 172 μV·K^−1^ at 800 K for the SPS-processed sample to 157 μV·K^−1^ for the HP-textured sample. This is slightly lower than the figures reported for a single crystal (210 μV·K^−1^) [36] or thin films (185 μV·K^−1^) [38].

**Figure 7:**
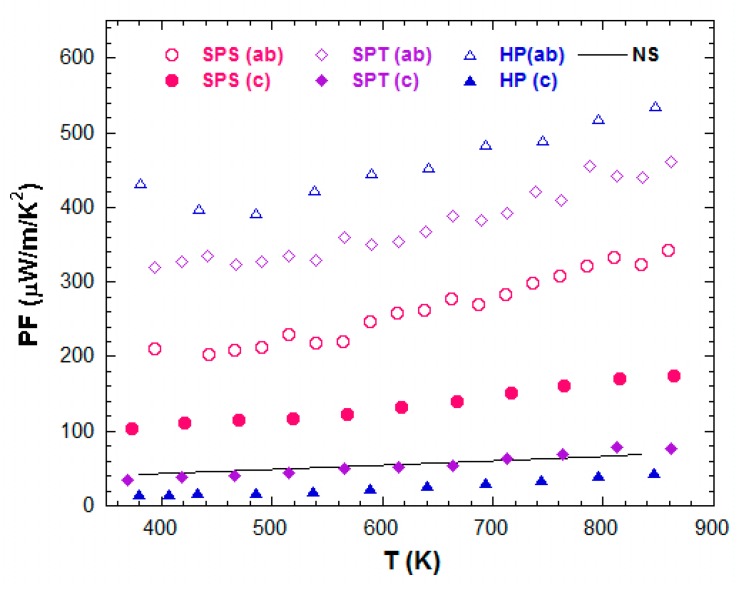
Temperature dependence of the resulting power factor (PF) along both principal directions in the SPS, SPT, and HP stacks. *PF* ^NS^ (T) obtained for the NS sample is also plotted for comparison.

Figure 7 shows the temperature dependence of the resulting power factor (PF = S^2^/*ρ*) along both principal directions in the SPS, SPT, and HP stacks. PF^ab^ was much larger than PF^c^ for all samples, reflecting the electrical resistivity dominance on the magnitude of PF on the one hand and on the strong anisotropy on the other hand. The PF anisotropy ranged *PF*^c^/*PF*^ab^ (_HP_) < *PF*^c^/*PF*^ab^ (_SPT_) < *PF*^c^/*PF*^ab^ (_SPS_). The *PF*^ab^/*PF*^c^ ratio reached 1.93, 6, and 13.6 at 800 K for the thick SPS, SPT, and HP samples, respectively. The latter value represents the largest achieved hitherto on Ca_3_Co_4_O_9_ ceramics [44,45]. Note that the *PF*^ab^ recorded for the SPT sample represented a 36% increase compared to the SPS. Such an improvement of PF, accompanied by the outstanding time and energy savings brought by the SPT process compared to HP, emphasizes the decisive benefits of the former in texturing lamellar materials like Ca_3_Co_4_O_9_. The *PF*^ab^ value (520 μW·m^−1^·K^−2^ at 800 K) noted for the HP sample was, however, among the largest ones reached so far on undoped Ca_3_Co_4_O_9_ ceramics [25,26,29,38], but remains 2 and 1.5 times lower compared to the reported single crystal [36] and thin films [38].

## 5. Conclusions

In this study, thick textured Ca_3_Co_4_O_9_ ceramics were successfully fabricated by moderately pressing at 1173 K stacks of pellets primarily textured using SPS, SPT, and HP processes. The latter two methods were both found to be effective for lamellar material texturing and (a,b) planes grain growth, inducing a GBD^ab^ drop and a GBD^c^ increase. Even though the HP technique enabled the strongest texture and the highest GBD^c^/GBD^ab^ ratio, the SPT technique represents decisive benefit of outstanding time and energy savings. Their respective *ρ*^ab^ values at 800 K were 6.7 and 5.3 mΩ·cm corresponding to ~6 and 8 times, respectively, lower than the *ρ*^ab^ of the naturally sintered sample (42 mΩ·cm). The resistivity anisotropy *ρ*^c^/*ρ*^ab^ as well as thermopower anisotropy S^c^/S^ab^ was demonstrated to be directly influenced by the texture strength and the GBD^c^/GBD^ab^ ratio; S^c^/S^ab^ being more influenced by grain boundaries anisotropy and *ρ*^c^/*ρ*^ab^ more influenced by texture. The power factor was also strongly anisotropic because of resistivity anisotropy and was found as *PF*^c^/*PF*^ab^ (_HP_) < *PF*^c^/*PF*^ab^ (_SPT_) < *PF*^c^/*PF*^ab^ (_SPS_). The *PF*^ab^ recorded for the SPT sample was 36% larger than for SPS, while that recorded for the HP-textured material was among the largest reached so far on undoped Ca_3_Co_4_O_9_ ceramics.
